# Xenogeneic Human p53 DNA Vaccination by Electroporation Breaks Immune Tolerance to Control Murine Tumors Expressing Mouse p53

**DOI:** 10.1371/journal.pone.0056912

**Published:** 2013-02-15

**Authors:** Ruey-Shyang Soong, Janson Trieu, Sung Yong Lee, Liangmei He, Ya-Chea Tsai, T.-C. Wu, Chien-Fu Hung

**Affiliations:** 1 Departments of Pathology, Johns Hopkins Medical Institutions, Baltimore, Maryland, United States of America; 2 Obstetrics and Gynecology, Johns Hopkins Medical Institutions, Baltimore, Maryland, United States of America; 3 Molecular Microbiology and Immunology, Johns Hopkins Medical Institutions, Baltimore, Maryland, United States of America; 4 Oncology, Johns Hopkins Medical Institutions, Baltimore, Maryland, United States of America; 5 Department of General Surgery, Chang Gung Memorial Hospital at Keelung, Keelung City, Taiwan; 6 Chang Gung University, College of Medicine, Taoyuan, Taiwan; 7 Johns Hopkins University, Baltimore, Maryland, United States of America; 8 Department of Internal Medicine, Korea University Medical Center, Seoul, South Korea; Istituto Superiore di Sanità, Italy

## Abstract

The pivotal role of p53 as a tumor suppressor protein is illustrated by the fact that this protein is found mutated in more than 50% of human cancers. In most cases, mutations in p53 greatly increase the otherwise short half-life of this protein in normal tissue and cause it to accumulate in the cytoplasm of tumors. The overexpression of mutated p53 in tumor cells makes p53 a potentially desirable target for the development of cancer immunotherapy. However, p53 protein represents an endogenous tumor-associated antigen (TAA). Immunization against a self-antigen is challenging because an antigen-specific immune response likely generates only low affinity antigen-specific CD8^+^ T-cells. This represents a bottleneck of tumor immunotherapy when targeting endogenous TAAs expressed by tumors. The objective of the current study is to develop a safe cancer immunotherapy using a naked DNA vaccine. The vaccine employs a xenogeneic p53 gene to break immune tolerance resulting in a potent therapeutic antitumor effect against tumors expressing mutated p53. Our study assessed the therapeutic antitumor effect after immunization with DNA encoding human p53 (hp53) or mouse p53 (mp53). Mice immunized with xenogeneic full length hp53 DNA plasmid intramuscularly followed by electroporation were protected against challenge with murine colon cancer MC38 while those immunized with mp53 DNA were not. In a therapeutic model, established MC38 tumors were also well controlled by treatment with hp53 DNA therapy in tumor bearing mice compared to mp53 DNA. Mice vaccinated with hp53 DNA plasmid also exhibited an increase in mp53-specific CD8^+^ T-cell precursors compared to vaccination with mp53 DNA. Antibody depletion experiments also demonstrated that CD8^+^ T-cells play crucial roles in the antitumor effects. This study showed intramuscular vaccination with xenogeneic p53 DNA vaccine followed by electroporation is capable of inducing potent antitumor effects against tumors expressing mutated p53 through CD8^+^ T cells.

## Introduction

The tumor suppressor protein p53, encoded by the TP53 gene in humans, is a necessary piece of the genome guarding mechanism [1] and is thus highly conserved. P53 serves to halt the cell cycle during normal instances of DNA damage allowing time for repair. However, for this very reason, p53 is frequently inactivated via sequence mutations in more than 50% of common human cancers [2]. Normally, in the absence of cell stressors, p53 is unstable and readily degraded in a process mediated by the ubiquitin ligase Mdm2. But, once mutated, p53 becomes upregulated and accumulates within the tumor cells. Consequently, p53 appears to be a promising target for clinical immunotherapy[3], [4]. However, as a tumor associated antigen (TAA), p53 proves to be poorly immunogenic. TAAs, in contrast to tumor specific antigens (TSA), are expressed both in tumor and normal cells. Therefore, attempts at immunization against TAAs generate, at most, low affinity antigen-specific CD8+ T-cells [5]–[7]. This issue remains a bottleneck for tumor immunotherapy.

Naked xenogeneic DNA vaccines, as the name suggests, incorporate plasmid DNA from a species foreign to the host in order to treat a disease. The DNA sequence utilized encodes for a gene homologous between the two species. There is often an optimal homology range in order for the vaccine to elicit an immune response. If the two sequences are too similar the immune system does not recognize the xenogeneic DNA as foreign. Once transfected into the patient's cells via clinical methods (intramuscular injection, gene gun, and electroporation), the mechanisms of transcription and translation produce a xenogeneic protein. The protein is processed and expressed on MHC class I molecules resulting in an immune response. Previous studies have proven the effectiveness of this method, most notably in the use of human tyrosinase DNA in treating canine malignant melanoma [8].

In the current study, we examined the immune response garnered from the intramuscular administration of a xenogeneic human p53 (hp53) naked DNA vaccine followed by electroporation. Electroporation is a common DNA vaccine administration technique utilized to great effect in our previous studies due to its potent generation of antigen-specific immune responses [9], [10]. We found that vaccinated C57BL/6 mice were successfully protected from tumor challenge the murine colon cancer cell line highly expressing mouse p53 (mp53), MC38. The xenogeneic naked DNA vaccine also proved effective in controlling established MC38 tumors. Furthermore, vaccinated C57BL/6 mice exhibited an increase in mp53-specific CD8+ T-cell precursors. Thus, xenogeneic p53 naked DNA vaccinations are a promising method for treating various forms of cancer. In addition, the method itself shows the potential to resolve the TAA issue of other diseases.

## Materials and Methods

### Ethics Statement

All animal procedures were performed according to approved protocols and in accordance with recommendations for the proper use and care of laboratory animals by Johns Hopkins University Animal Care and Use Committee.

### Mice

6- to 8-week-old female C57BL/6 mice were purchased from the National Cancer Institute-Frederick Animal Production Area (Frederick, MD) and housed in the oncology animal facility of the Johns Hopkins Hospital (Baltimore, MD).

### Cell Lines

MC38 is a murine colon adenocarcinoma cell line generated from C57BL/6 mice that highly expresses mouse p53 protein [11], [12]. Cells were cultured in RPMI1640 medium containing 10%FBS, 2 mM L-glutamine, 10% sodium pyruvate, 10% non-essential amino acid, and 100 pg/ml streptomycin in humidified atmosphere of 5%CO2/95% air at 37°C. Luciferase- and GFP-expressing MC38 (MC38-GFP/Luc) was generated by transduction with a lentivirus containing luciferase and GFP using lentiviral vector pCDH1-luc-EF1-GFP as previously described [13]. RMA, a mouse lymphoma cell line without p53 expression [14], was also transduced GFP and luciferase.

### DNA constructs

Human p53 DNA (pCMV-hp53) was purchased from Addgene (Cambridge, MA). To generate pcDNA3-mp53 (mouse p53), mp53 was first amplified with PCR using pMXs-53 (Addgene) as a template and the following set of primers: 5'- TTTGAATTCACCGCCATGACTGCCATGGAGGAGTCAC-3'and 5'-AAAGGATCCTCAGTCTGAGTCAGGCCCCACTT'-3'. The PCR product was cloned into EcoRI and BamHI sites of the pcDNA3.1(-) vector. To generate pET28a-mp53, mp53 was first amplified by PCR using pMXs-53 as a template and the following set of primers: 5'- AAAGGATCCATGACTGCCATGGAGGAGTC -3'and 5'- TTTGAATTCGTCTGAGTCAGGCCCCACTT'-3'. The PCR product was cloned into the BamHI and EcoRI sites of the pET28a vector. A lentiviral construct pCDH-Luc-EF1-GFP (System Biosciences, Mountain View, CA, USA) was also used, which expresses both luciferase and GFP.

### Mouse p53 protein purification

The pET28a-mp53 was transformed and expressed in E. coli BL21 (Rosetta cells; Novagen). The selected colony was cultured in 5 mL Luria–Bertani (LB) liquid medium containing kanamycin (25 ug/mL) and grown overnight at 37°C on a shaking incubator, then transferred to 200 mL of fresh medium (with the antibiotic) and incubated for another 2 hours until the optical density (OD600) of the cultured cells reached approximately 0.6. Expression of the fusion protein was induced with 1 mM isopropyl-b-D-thiogalactopyranoside (IPTG) at 37°C for 5 h. The cultured cells were harvested by centrifugation at 6,000 rpm for 10 min at 4°C. The pellet was washed twice with PBS and then suspended in bacteria lysis buffer (SoluLyse Reagent for Bacteria, Genlantis) containing lysozyme (100 ug/ml) (Gibco BRL) and Dnase I (100 U/ml)(Invitrogen). The suspension was incubated for 2 hours at room temperature with stirring. The suspension was centrifuged at 12,000 rpm for 15 min. The clear supernatant (soluble fraction) was collected and recombinant protein was purified by Ni^+^ affinity chromatography (Ni-NTA agarose, Qiagen) according to the manufacturer's protocol. Briefly, cell supernatant was loaded in 2 ml of Ni^+^ affinity chromatography that is equilibrated with washing buffer (50 mM NaH_2_PO_4_, 300 mM NaCl, and 20 mM imidazole, pH 8.0) and then washed with 20 ml washing buffer. For the elution of binding protein, 10 ml of elution buffer (50 mM NaH_2_PO_4_, 300 mM NaCl, and 250 mM imidazole, pH 8.0) was used. The eluted protein was collected and purity was analyzed using 10–15% gradient SDS–PAGE and Coomassie brilliant blue staining (Figure S1).

### Electroporation-mediated DNA vaccination

The square-wave electroporator (Model 830; BTX) consists of an electrode array, a pulse generator, and a foot pedal for pulse activation. Disposable 30G needles (Becton-Dickinson, Franklin Lakes, NJ) were used in the center of the electrode grid to administer DNA plasmid equidistant from the two needle array electrodes (BTX, San Diego, CA). Mice were injected in the tibialis muscle of the shaved hind leg with activation of the pulse generator as instructed by the manufacturer and as previously described[9]. 10 µg of DNA plasmid was diluted in a total volume of 20 µL of PBS for each injection. When vaccination schedules required a booster vaccination, the contralateral leg was used for vaccination. Subsequent vaccinations were administered on alternating hind legs.

### Detection of mp53-specific antibodies in the serum of vaccinated mice by ELISA and Western blot

The presence of anti-mouse p53 antibodies in the sera was characterized by a direct ELISA as described previously [15]. A cohort of mice was immunized with 10 µg of pCMV vector, pCDNA3-mp53, or pCMV-hp53 DNA vaccine by electroporation and received two booster vaccinations every 7 days. Sera were prepared from mice 7 days after the last vaccination. The ELISA plate was read with a standard ELISA reader at 450 nm using serial dilutions of prepared sera. Western blot analysis was performed with 10 µg of mp53 protein and sera from each vaccinated mouse followed by anti-mouse mp53 Ab and anti-HA mouse mAb (clone 12CA5; Roche Diagnostics Corp., Indianapolis, Indiana) using methods described previously [16].

### Intracellular cytokine staining with cytometric analysis to detect IFN-γ secretion by p53-specific CD8^+^ T cells

Cell surface marker staining for CD8 and intracellular cytokine staining for IFN-γ as well as FACScan analysis were performed in the same conditions as previously mentioned [17]. Three mice in each group were vaccinated with 10 µg of pCMV vector, pCDNA3-mp53, or pCMV-hp53 DNA vaccine by electroporation and received two booster vaccinations every 7 days. One week after the last vaccination, splenocytes (2×10^6^) were collected and incubated with 1 ug/ml of p53 peptide 232 (KYMCNSSCM) as previously mentioned [18] in 24-well plates under IL-2 (20 IU/ml) stimulation for one week as previously mentioned. The numbers of IFN-γ-secreting CD8^+^ T cells were analyzed by FACScan cytometry. Analysis was performed on a Becton-Dickison FACScan with CELLQuest software and flowjo software (Becton-Dickinson Immunocytometry System, Moutain View, CA).

### *In vivo* tumor protection

For all tumor experiments, MC38 murine colon adenocarcinoma cell line was used. Four or five mice per group were vaccinated with 10 µg of pCMV vector, pCDNA3-mp53, or pCMV-hp53 DNA vaccine via electroporation followed by three weekly booster vaccinations using the same method. One week after the last vaccination each mouse was challenged with 2×10^5^ MC38 cells injected subcutaneously. All mice were observed for 80 days to monitor tumor progression. Tumor growth was determined by electronic caliper twice a week and the percentages of surviving mice were recorded. Tumor volume is approximated by the following formula: length x width^2^×0.5. Animals showing severe distress or bearing tumors that exceed 1.5 cm in diameter are euthanized in accordance with animal care protocol.

### *In vivo* tumor treatment

Five mice per group were challenged subcutaneously with 2×10^5^ MC38 cells/mice. Five days after tumor challenge, mice were vaccinated with 10 µg of pCMV vector, pCDNA3-mp53, or pCMV-hp53 DNA via electroporation. Mice received a booster vaccination at 5-day intervals for a total of three vaccinations in alternating hind legs. Tumor size was measured with electronic calipers and tumor volume calculated starting on day 6. Measurements were recorded twice per week.

### *In vivo* antibody-depletion experiment

Five mice in each group were vaccinated with pCMV-hp53 DNA vaccine as previously mentioned. After the last vaccination, CD4, CD8, and NK1.1 depletion (100 µg/mice) was initiated at 2 day intervals one week before tumor challenge. After tumor challenge, antibody was given every 7 days until day 42. Tumor growth was determined by electronic calipers twice a week and the percentages of surviving mice were recorded.

### Cytolytic activity assay

A cytolytic activity assay utilizing luciferase to measure target cell viability was previously described [19]. MC38-GFP-Luc cells and RMA-GFP-Luc cells were added to 96-well plates at 5×10^4^ cells/well and incubated for 6 hours. Next, mP53-specific T cells from different vaccinated groups were added in titrated ratio (100∶1, 50∶1, 20∶1,10∶1 and control) to each well for 12 hours at 37°C. The wells were washed twice with PBS before coelenterazine was added. Bioluminescence of the cells was detected via the IVIS Imaging System 200 Series. The region of interest from displayed images was designated and quantified as total photon counts using Living Image 3.2 software (Xenogen).

### Statistical analysis

All data are expressed as mean ± SE where indicated. Comparisons between individual data points for intracellular cytokine staining with flow cytometric analysis and tumor treatment were made using Student's t-test. In the tumor protection experiments, the principal outcome of interest was duration until development of a tumor >1.0035 cm. The event-time distributions for different mice were compared using the Kaplan–Meier method and the log-rank statistic by SPSS 17 software. All p-values <0.05 were considered significant.

## Results

### Xenogeneic p53 vaccination generates higher levels of circulating mp53-specific antibody responses compared with other vaccinations

**Figure 1:**
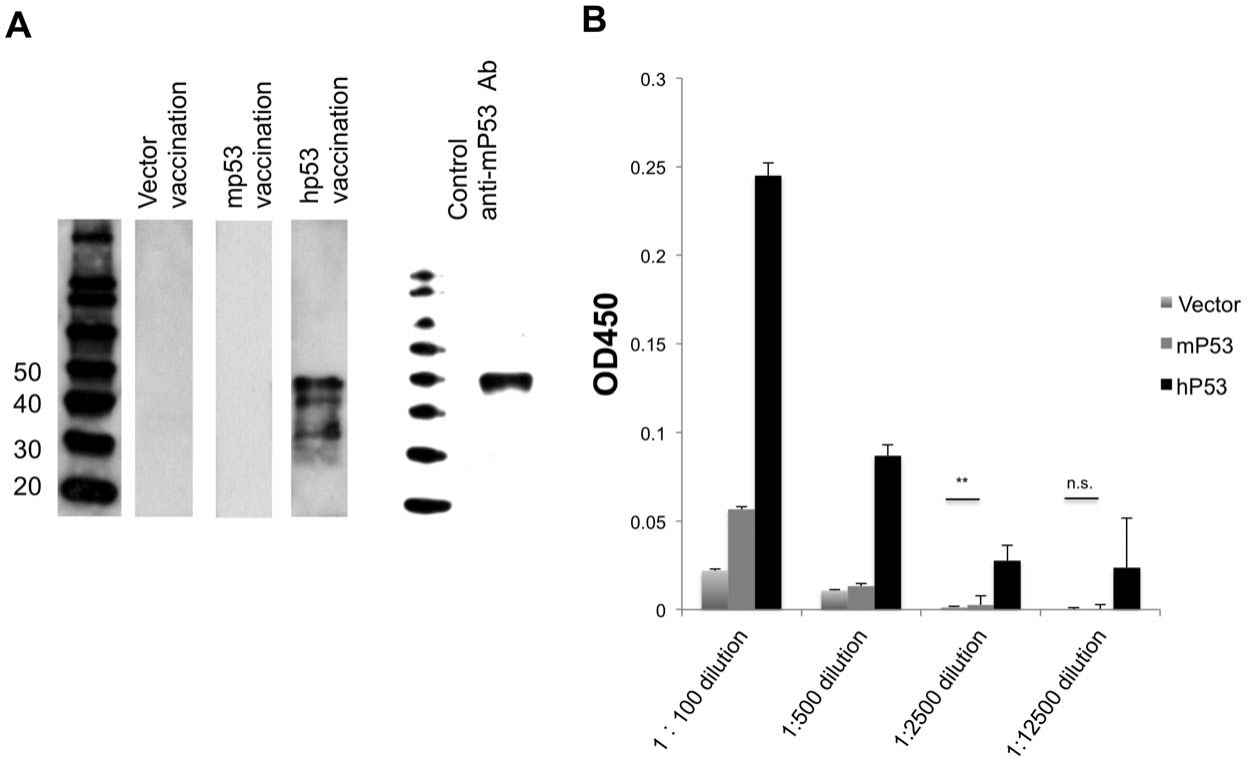
Mice vaccinated with hp53 DNA generated strong antibody responses to mp53 protein. (A) Serum from vector, mp53, and hp53 vaccinated mice showed different antibody responses to p53 protein on the Western blot. (B) ELISA demonstrates p53-specific Ab's in mice vaccinated with various DNA vaccines. The result shows the sequential dilution 1∶100, 1∶500, 1∶2500, and 1∶12500, detected by mean absorbance (OD450 nm) ± S.E. N = 3 per group (*, P<0.05).

A cohort of mice (three per group) was vaccinated with vector only, mp53, or hp53 DNA plasmid vaccine by electroporation. Subsequently, mice received a booster vaccination in alternating hind legs every 7 days for a total of three vaccinations. Sera from these mice were collected 14 days after the last vaccination and analyzed by Western blot (Fig. 1A). Western blot revealed that sera from hp53 vaccinated mice showed a strong signal against mp53 protein whereas sera from mp53 vaccinated mice did not.

We subsequently used ELISA to analyze the antibody binding affinity for the mp53 protein. Figure 1B shows serial dilutions of the sera from vaccinated mice. The assay revealed that hp53 vaccination generated significantly higher levels of mp53-specific antibodies up to 1∶2500 dilution (0.02, p<0.05).

### hp53 DNA vaccine generates the greatest level of IFN-γ-producing CD8^+^ T cells

**Figure 2:**
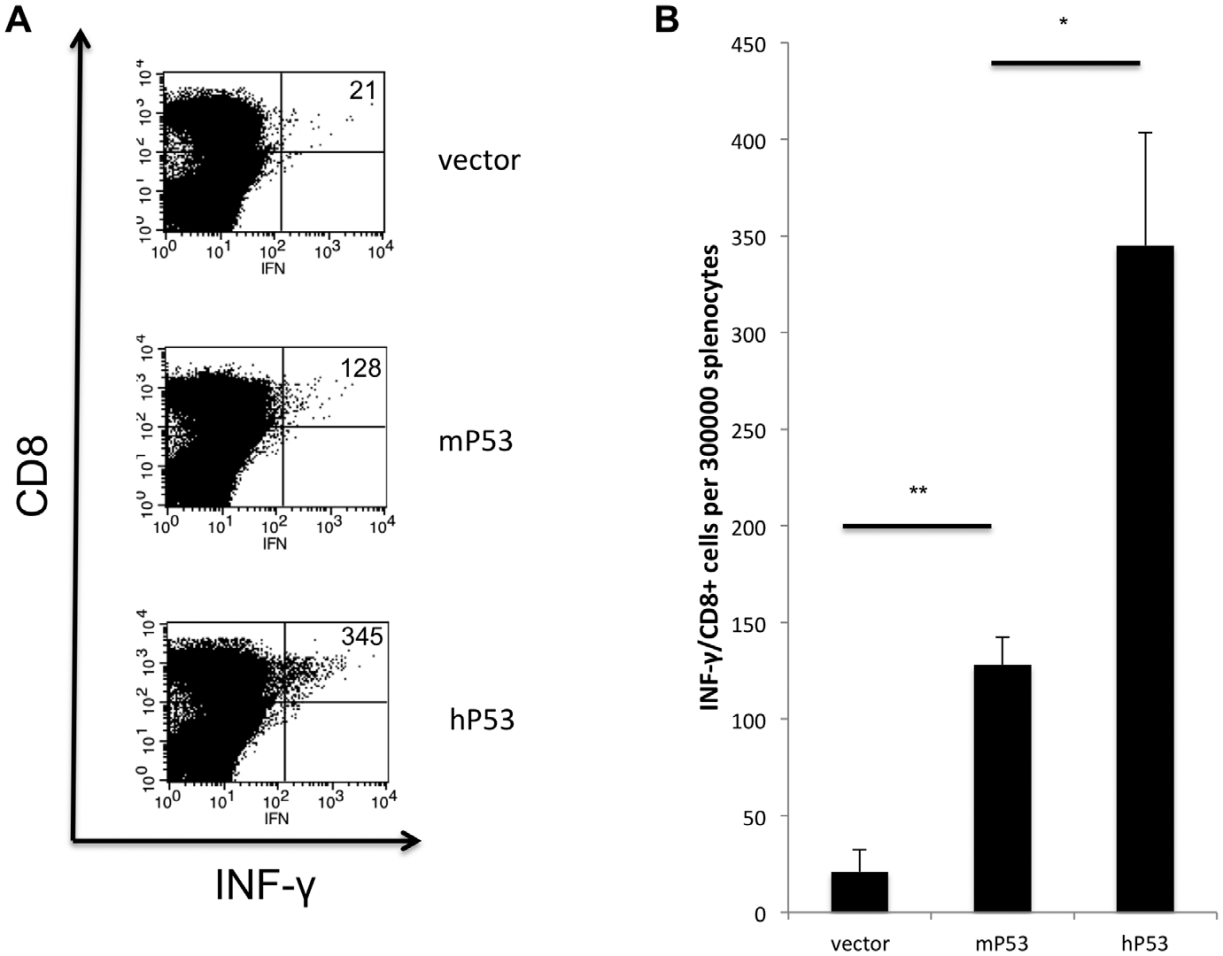
Mice vaccinated with hp53 DNA had the highest CD8 T cell activation. Splenocytes from vaccinated mice were cultured with p53 epitope 232 under IL-2 stimulation for one week (A) (B) Mice vaccinated with hp53 DNA vaccine generate 345±58.6 (mean ± S.E) IFN-γ/CD8+ cells per 30**0**,000 splenocytes, comparing to mp53 vaccination group was 128±14.4. N = 3 for each group (*, P<0.05 **, P<0.01).

We then compared the ability of different vaccines to elicit INF-γ-producing CD8^+^ T cells by performing intracellular cytokine staining on splenocytes obtained from treated mice. DNA plasmids containing hP53, mP53 or control vector were administered to each group of mice by intramuscular injection followed by electroporation at one-week intervals, for a total of three vaccinations. Seven days after the last vaccination, splenocytes from each group were incubated with mp53 peptide 232 under IL-2 stimulation for one week. Figure 2 shows that mice treated with pCMV-hp53 DNA plasmid vaccine generated higher numbers of IFN-γ-producing CD8+ T cells (345±58.6/300,000 splenocytes) than those in the pCDNA3-mp53 DNA vaccine group (128±14.4/300,000 splenocytes). Our data indicate that mp53-specific CD8+ T cells generated by DNA vaccines were significantly enhanced by administration with hp53 DNA plasmid.

### Mice vaccinated with the hp53 DNA vaccine displayed superior protection against murine MC38 tumors

**Figure 3:**
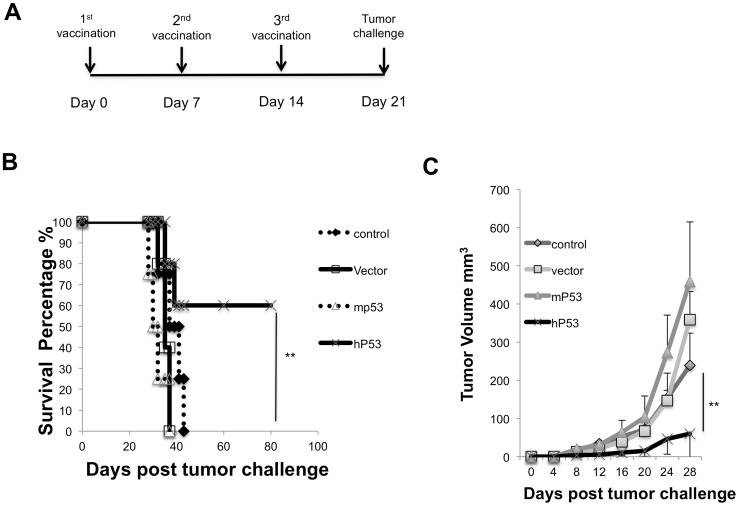
*In vivo* tumor protection experiments in mice vaccinated with various DNA vaccines. (A) Vaccination schedule. (B) Mice (n = 5) were immunized with various DNA vaccines and challenged with MC38 cells (2×10^5^) to assess the survival curve by each DNA vaccine. C) Tumor volume was measured weekly with digital calipers. Data are expressed as volume ± S.E. N = 5 in each group (**, P<0.01).

Briefly, C57BL/6 mice (four or five in each group) were vaccinated three times with a vector only, pCDNA3-mP53, or pCMV-hP53 DNA plasmid via electroporation in accordance with the schedule outlined in Figure 3A. On day 21 following the first vaccination, 2×10^5^ MC38 tumor cells were inoculated subcutaneously and were closely observed for palpable tumor development. As shown in Figures 3B and 3C, mice vaccinated with pCMV-hP53 had a significantly higher survival rate and decreased tumor volume compared to pCDNA3-mP53 vaccinated mice (p<0.01). After 80 days post tumor challenge, 60% of the pCMV-hP53 vaccinated mice were tumor free.

### hp53 DNA vaccine induces a positive therapeutic effect in MC38 tumor bearing mice

**Figure 4:**
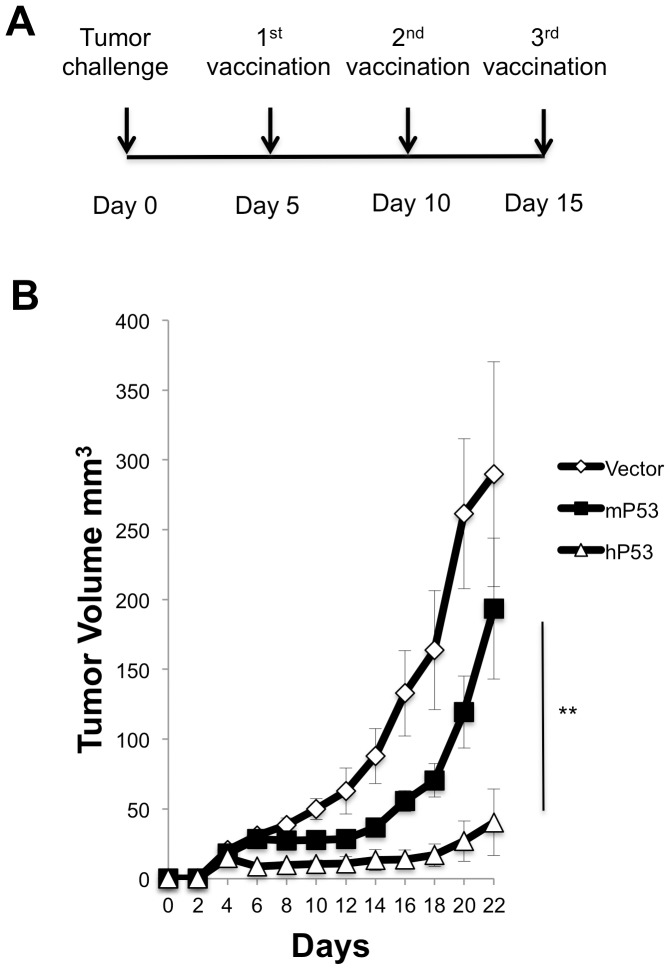
*In vivo* therapeutic vaccination. (A) Therapeutic schedule. (B) Mice (n = 5) were inoculated s.c. with **2**×10^5^ MC38 cells. Mice were immunized using either vector only, mp53, or hp53 DNA vaccine administered via injection with electroporation, starting on day 5 at 5 day intervals. Tumors were measured with digital calipers and tumor volumes were calculated. Data are expressed as volume ± S.E. N = 5 in each group (**, P<0.01).

The most stringent model of vaccine efficacy is *in vivo* activity against a growing tumor. Using this model, we compared the different DNA vaccines against an MC38 tumor cell line. Briefly, mice (five in each group) were injected with 2×10^5^ MC38 tumor cells subcutaneously on Day 0 (Fig. 4A). Mice were subsequently vaccinated on day 5, day 10 and day 15 with 10 µg of vector only, pCDNA3-mp53, or pCMV-hp53 DNA plasmid via electroporation. Tumor volume was measured twice a week. The data revealed that mice vaccinated with pCMV-hp53 elicited a sustained anti-tumor response compared with the pCDNA3mp53 and vector only vaccinated groups (Fig. 4B).

### Tumor protection effect in hp53 vaccinated mice was lost following CD8^+^ T cell depletion via anti-CD8 mAb

**Figure 5:**
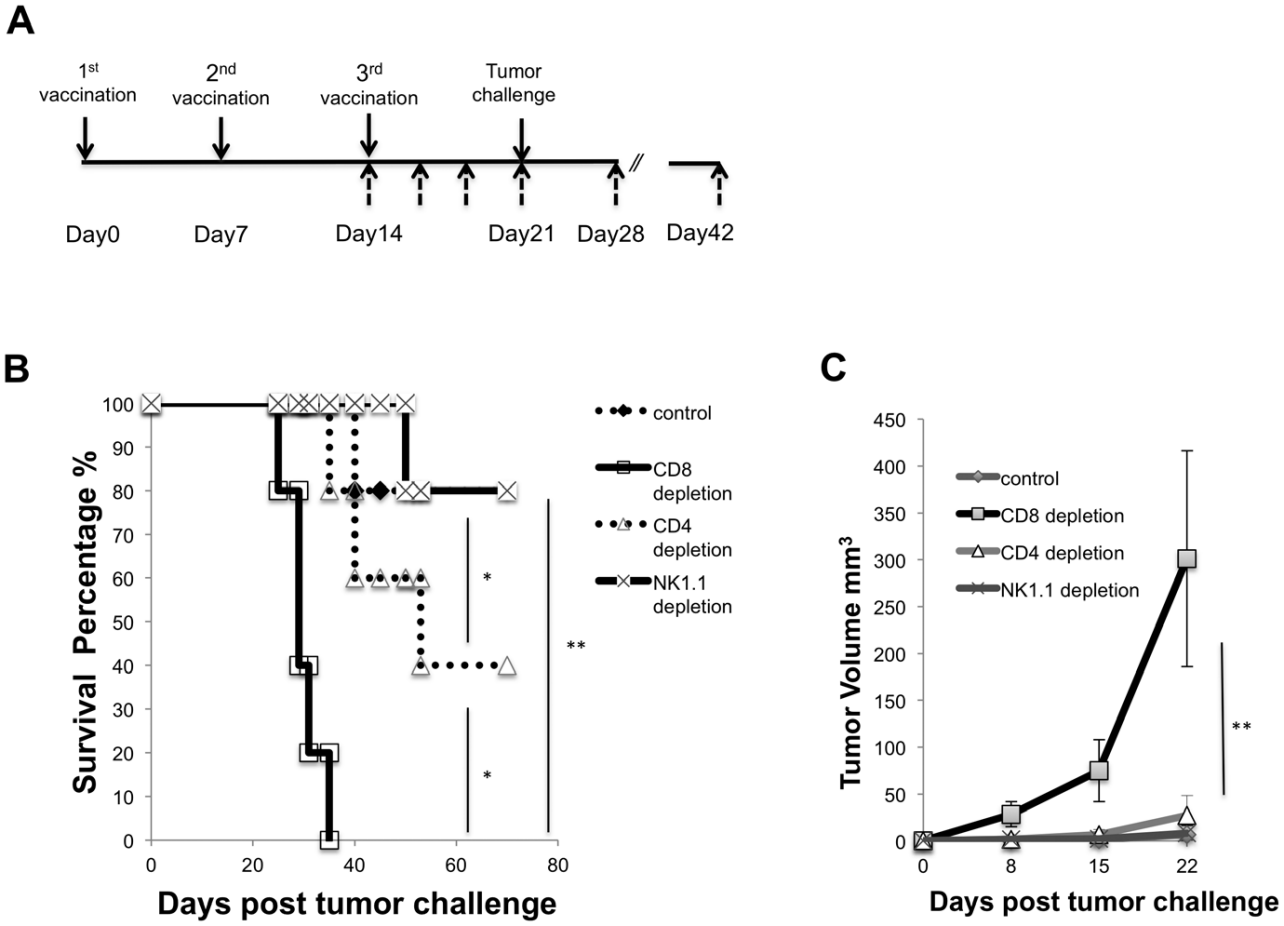
*In vivo* antibody depletion experiments in mice vaccinated with hp53 DNA plasmid. (A) Therapeutic schedule. (B) Mice (n = 5) were immunized by hp53 DNA vaccine administered via injection with electroporation and challenged with **2**×10^5^ MC38 cells to determine the effect of lymphocyte subsets on the potency of the hp53 DNA vaccine. CD4, CD8, and NK1.1 depletions were initiated one week before tumor challenge and lasted 30 days after tumor challenge, with treatments at one day intervals. (C) Tumor volume was measured weekly with digital calipers. Data are expressed as volume ± S.E. N = 5 in each group**.** N = 5 in each group (*, P<0.05 **, P<0.01).

Next, we were eager to discover which lymphocytic group was responsible for the tumor protection effect after hp53 plasmid DNA vaccination. Mice (five per group) were vaccinated with pCMV-hp53 plasmid DNA using electroporation (Figure 5A) for a total of three vaccinations. After the last vaccination, anti-CD4, anti-CD8 or anti-NK1.1 mAb (100 µg/mice) were injected intraperitoneally every other day. On day 28, MC38 tumor cells (2×10^5^) were inoculated subcutaneously and the tumor volume was measured twice a week. Figures 5B and 5C reveal that after CD8+ T cell depletion, mice had significantly larger tumor volume and poorer survival compared to the other three treatment groups. This suggests that CD8+ T cells are important for the antitumor immunity generated by the hp53 vaccine.

### CD8+ T cells vaccinated with hP53 enhanced antigen specific killing compared to those vaccinated with mP53 in vitro

**Figure 6:**
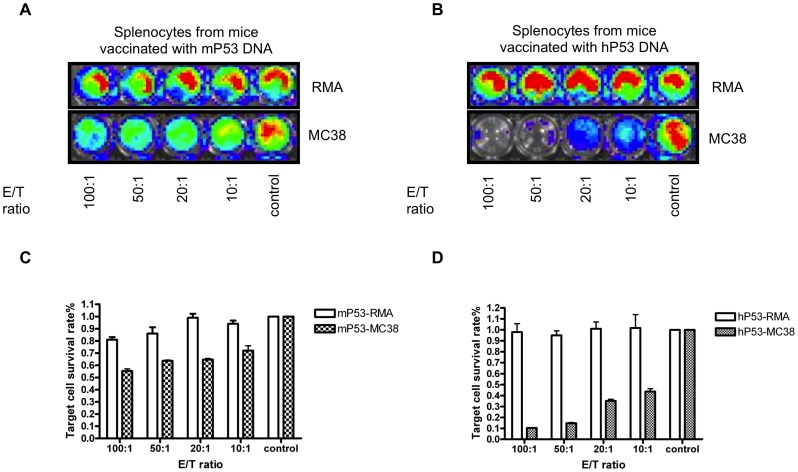
*In vitro* p53 specific T cell killing assay detection by luciferase bioluminescence imaging system. (A, B) C57/BL6 mice (5 per group) were vaccinated with DNA encoding mP53 or hP53. Splenocytes from vaccinated mice were isolated and stimulated in vitro with mP53-specifc CTL epitope (KYMCNSSCM) and IL-2. Splenocytes were then incubated with RMA-GFP-Luc or MC38-GFP-Luc cell line (5×10^4^ cells/well) in different E/T ratios. The luciferase signal of live tumor cells was detected by IVIS imaging system. (C, D) Control group was set as 100% survival rate of tumor cells. Luciferase activity correlates with survival of tumor cells (*P<0.01).

In order to show the vaccinated CD8 T cells are antigen specific, we utilized an in vitro T cell killing assay employing a luciferase-based bioluminescence imaging system. Briefly, splenocytes from mice vaccinated with DNA encoding mP53 and hP53 were harvested after vaccination four times and treated by in vitro stimulation with mp53 CTL epitope, peptide 232, in conjunction with IL-2 for two cycles. The T cells were subsequently added into pre-seeding MC38-GFP-Luc and RMA-GFP-Luc by titrated effector to target cell ratio (E/T ratio). As shown in Figure 6, while the T cells from mP53 and hP53 DNA vaccinated mice show limited killing function of RMA-GFP-Luc cells, which does not express p53, T cells from hP53 DNA vaccine treated mice demonstrated significantly enhanced killing of MC38-GFP-Luc cells compared to T cells from mP53 DNA vaccinated mice. This result indicates that mice vaccinated with DNA encoding hP53 are able to generate mP53-antigen specific CD8+ T cells that are capable of killing mP53-expressing tumor cells.

## Discussion

In the current study, we determined that vaccination utilizing a xenogeneic hp53 naked DNA vaccine via intramuscular injection followed by electroporation generated a robust antibody response against mp53. These anti-mp53 antibodies were found to be both highly abundant and specific in comparison to vector and mp53 controls (Fig. 1). In addition, the vaccine assisted in mp53-specific CD8+ T-cell proliferation and activation (Fig. 2). Furthermore, we identified these CD8+ T-cells as the major instigators of the strong prophylactic and therapeutic antitumor effects observed in mice challenged with mp53 and expressing murine colon cancer MC38 tumor cells (Fig. 3, 4, and 5). Therefore, administration of xenogeneic naked DNA via intramuscular injection followed by electroporation bypasses the tolerance barrier enabling the successful use of TAAs as targets for immunotherapy. Thus, this method has a wide range of potential applications in treating diseases with similar tolerance issues.

Such a strategy has already proven successful as seen in the case of ONCEPT^TM^ Canine Melanoma (CMM) Vaccine licensed by Merial. As of 2010, ONCEPT^TM^ has been approved by the USDA to treat CMM, which represents approximately 4% of all canine tumors. The vaccine utilizes xenogeneic plasmid DNA encoding human tyrosinase to initiate an immune response against the TAA tyrosinase [8]. Survival time in CMM afflicted canines receiving standardized therapy was 1-5 months. Median survival time in canines with advanced CMM was approximately one year while the study was conducted [8], [18]. There are also some xenogeneic vaccines using different tumor antigens in mouse models that support this concept, such as telomerase reverse transcriptase [20], human N′-terminal neu DNA vaccine [21], human tumor endothelial marker 8 DNA vaccine [22], and prostatic acid phosphatase dendritic cell based vaccine [23]. Based on our understanding, this is the first successful study targeting p53 antigens via a xenogeneic strategy.

In our approach, we provide proof of concept on a wider scale. Our target TAA p53 is applicable to an extensive range of diseases. For instance, considering only cancers, mutant p53 is upregulated and overexpressed in more than 50% of cancers afflicting humans. In comparison, tyrosinase is an oxidase that catalyzes the production of melanin. Thus, the antigen is highly specific to melanomas. In addition, p53 is implicated in animal cancers as seen in the predominant use of animal models in determining the protein's function. Nevertheless, these results require translation from animal models to clinical applicability. This transition necessitates the determination of a p53 gene that is xenogeneic to humans in order to target hp53. Still, our study validates the viability of xenogeneic naked DNA vaccines to be clinically applied as a more comprehensive treatment strategy. In summary, immunotherapies targeting p53 by xenogeneic strategy are potentially an effective method to break the tolerance bottleneck and control variable tumors highly expressing p53.

## Supporting Information

Figure S1 **Purification of p53 protein. mp53 protein purified from bacteria transfected with a plasmid containing mp53 DNA was verified by Coomassie brilliant blue staining.** Protein with molecular weight between 50∼75 kDa is shown in the SDS-PAGE gel staining with Coomassie blue.
(TIF)
