# Supplementary figures and images for: Xenogeneic Human p53 DNA Vaccination by Electroporation Breaks Immune Tolerance to Control Murine Tumors Expressing Mouse p53
Source: PLoS One. 2013 Feb 15;8(2):e56912. doi: 10.1371/journal.pone.0056912 (PMC3574113; doi:10.1371/journal.pone.0056912)

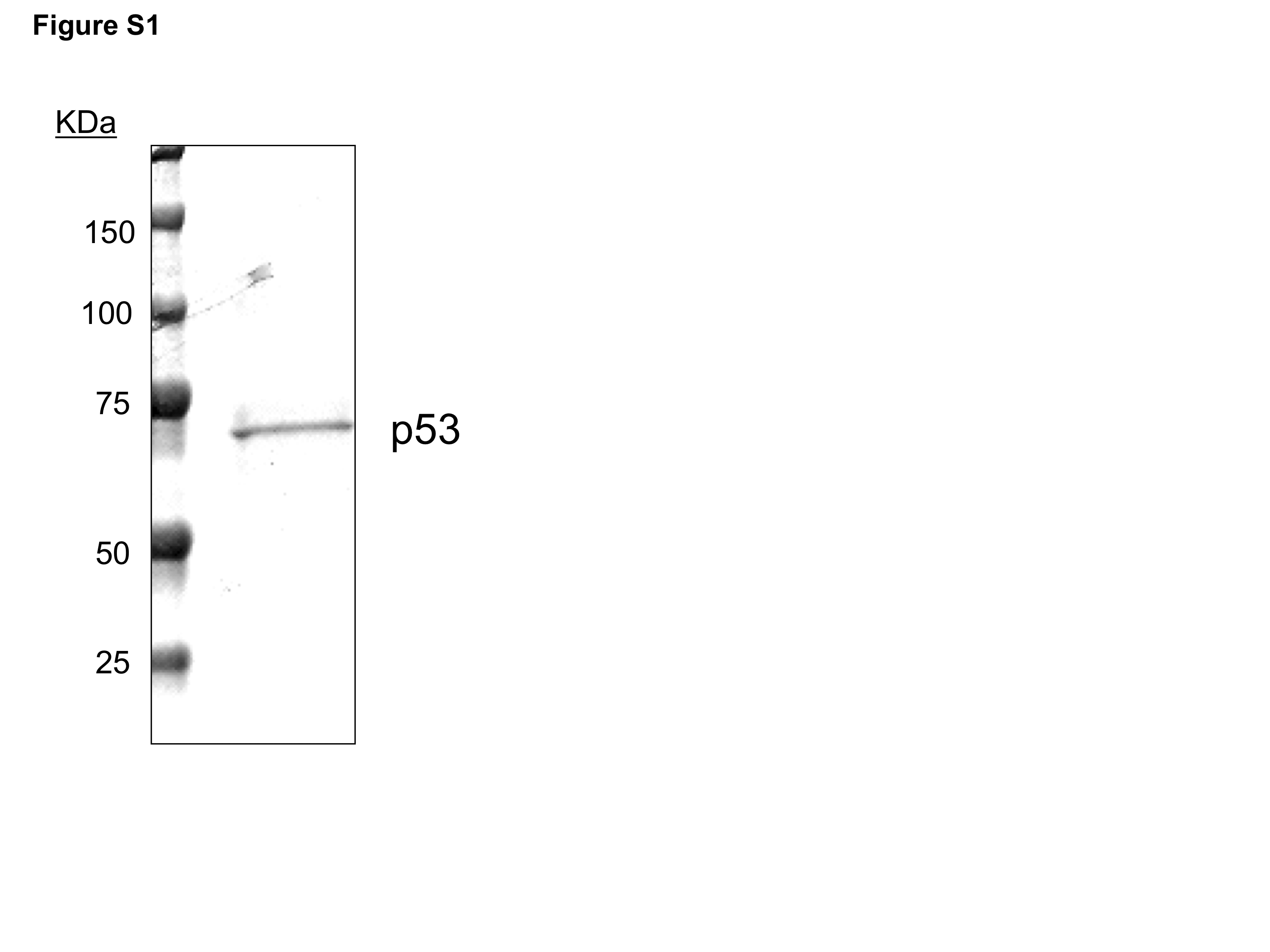

Supplement: Figure S1 — Purification of p53 protein. mp53 protein purified from bacteria transfected with a plasmid containing mp53 DNA was verified by Coomassie brilliant blue staining. Protein with molecular weight between 50∼75 kDa is shown in the SDS-PAGE gel staining with Coomassie blue. (TIF) [file pone.0056912.s001.tif]
